# Breast cancer cell-associated endopeptidase EC 24.11 modulates proliferative response to bombesin

**DOI:** 10.1038/sj.bjc.6690036

**Published:** 1999-01

**Authors:** D M Burns, B Walker, J Gray, J Nelson

**Affiliations:** School of Biomedical Sciences, University of Ulster at Jordanstown, Shore Rd, Newtownabbey, Co. Antrim, BT37 0QB, Northern Ireland; Division of Biochemistry, School of Biology and Biochemistry, Queen's University, Medical Biology Centre, 97 Lisburn Road, Belfast, BT7 1NL, Northern Ireland

**Keywords:** enkephalinase, bombesin, gastrin-releasing peptide, breast cancer cells, neutral endopeptidase 24.11

## Abstract

We have investigated the production, growth and inactivation of gastrin-releasing peptide (GRP)-like peptides in human breast cancer cell lines. Radioimmunoassay detected GRP-like immunoreactivity (GRP-LI) in T47D breast cancer cells but not in the conditioned medium, indicating rapid clearance. No GRP-LI was found in the ZR-75-1 or MDA-MB-436 cells or their conditioned medium. High-performance liquid chromatography (HPLC) analysis of the GRP-LI in the T47D cells revealed a major peak, which co-eluted with GRP_18–27_, and a minor more hydrophilic peak. In vitro stimulation of T47D cell growth by bombesin (BN) was enhanced to 138% of control levels (bombesin alone) by the addition of the selective endopeptidase EC 3.4.24.11 inhibitor phosphoramidon (0.1 ng ml^−;1^). Fluorogenic analysis using whole cells confirmed low levels of this phosphoramidon-sensitive enzyme on the T47D cells. This enzyme, previously unreported in human breast cancer cells, significantly modulates both T47D growth and its response to BN-induced growth. © 1999 Cancer Research Campaign


*British Journal of Cancer* (1999) **79**(2), 214–220
© 1999 Cancer Research Campaign

### Breast cancer cell-associated endopeptidase EC 24.11 modulates proliferative response to bombesin

**DM Burns[1], B Walker[2], J Gray[2] and J Nelson[2]**

[1]School of Biomedical Sciences, University of Ulster at Jordanstown, Shore Rd, Newtownabbey, Co. Antrim, BT37 0QB, Northern Ireland; [2]Division of Biochemistry, School of Biology and Biochemistry, Queen's University, Medical Biology Centre, 97 Lisburn Road, Belfast BT7 1NL, Northern Ireland

**Summary** We have investigated the production, growth and inactivation of gastrin-releasing peptide (GRP)-like peptides in human breast cancer cell lines. Radioimmunoassay detected GRP-like immunoreactivity (GRP-LI) in T47D breast cancer cells but not in the conditioned medium, indicating rapid clearance. No GRP-LI was found in the ZR-75-1 or MDA-MB-436 cells or their conditioned medium. High-performance liquid chromatography (HPLC) analysis of the GRP-LI in the T47D cells revealed a major peak, which co-eluted with $GRP_{18–27}$, and a minor more hydrophilic peak. In vitro stimulation of T47D cell growth by bombesin (BN) was enhanced to 138% of control levels (bombesin alone) by the addition of the selective endopeptidase EC 3.4.24.11 inhibitor phosphoramidon (0.1 ng $ml^{-1}$). Fluorogenic analysis using whole cells confirmed low levels of this phosphoramidon-sensitive enzyme on the T47D cells. This enzyme, previously unreported in human breast cancer cells, significantly modulates both T47D growth and its response to BN-induced growth.

**Keywords:** enkephalinase; bombesin; gastrin-releasing peptide; breast cancer cells; neutral endopeptidase 24.11

Bombesin (BN), a tetradecapeptide originally isolated from amphibian skin in 1970, has potent hypertensive and gastrin-releasing effects in mammals (Erspamer et al, 1970; Erspamer et al, 1972*a*,*b*). Its homologue, gastrin-releasing peptide (GRP), is a 27-amino-acid peptide (McDonald et al, 1979) that is highly conserved in amphibian, avian, canine, rat and human species (McDonald et al, 1980; Reeve et al, 1983; Orloff et al, 1984; Zoeller et al, 1989; Nagalla et al, 1992). A fully active decapeptide identical to the C-terminus of GRP ($GRP_{18–27}$) has also been isolated from porcine and human tissue (Minamino et al, 1984; Orloff et al, 1984). GRP-like immunoreactivity (GRP-LI) and receptor binding is distributed predominantly in the brain, spinal cord, gut and lung, where it is thought to act both as a growth factor and as a neuroregulatory hormone (Moody et al, 1980; Sunday et al, 1988).

GRP has been detected in 60–70% of small-cell lung cancers (SCLC) (Wood et al, 1981; Yamaguchi et al, 1983; Bostwick et al, 1984), and GRP receptors have been demonstrated in numerous SCLC cell lines (Moody et al, 1983). In addition, the involvement of GRP as an autocrine growth factor in SCLC is well established (Cuttitta et al, 1985). This prompted a vast research effort to develop GRP receptor antagonists as a potential treatment for SCLC. More recently, evidence has been accumulating to suggest that GRP may play a role in the growth of breast cancer cells. GRP-LI has been detected in a proportion of breast tumours (Foster and Tan, 1984; Gaudino et al, 1986; McKillop et al, 1988) but not in normal tissues (Bostwick and Bensch, 1985; Gaudino et al, 1986). Using Northern blotting, GRP mRNA has been detected in 25% of unselected primary breast tumours (Pagini et al, 1991). More recently, Vangsted et al (1991) detected GRP-LI in 39% of primary breast tumours from post-menopausal women. GRP-LI has been detected in a number of established breast cancer cell lines accompanied by much higher levels of the C-terminal extension peptide (CTEP) (Weber et al, 1989; Vangsted et al, 1991; Yano et al, 1992). GRP receptors have been described in breast cancer cell lines T47D and MDA-MB-436 (Giacchetti et al, 1990). Previously, we have shown that both BN and GRP stimulate proliferation of breast cancer cell lines when culture serum has been stripped of endogenous GRP-like peptides (Nelson et al, 1991); other workers have confirmed these findings (Yano et al, 1992).

The cell-surface enzyme neutral endopeptidase EC 24.11 (EC 3.4.24.11), known to be involved in the catabolism and inactivation of a number of neuropeptides in the brain and GI tract (Relton et al, 1983; Matsas et al, 1984), has also been shown to hydrolyse and inactivate GRP (Bunnett et al, 1988). This enzyme inactivates GRP and $GRP_{18–27}$ by cleaving the His25–Leu26 bond; the His20–Trp21 bond is also cleaved, but to a much lesser extent (Bunnett et al, 1988). Other work suggests that high levels of neutral endopeptidase EC 24.11 regulate GRP-mediated growth of fetal lungs (King et al, 1993), and there is also considerable evidence that this enzyme regulates extracellular GRP levels in SCLC cell lines. Colony formation in SCLC cell lines that secrete GRP-like peptides is potentiated when neutral endopeptidase EC 24.11 is inhibited using phosphoramidon. This increase in colony formation is reduced in the presence of GRP receptor antagonists, indicating that a major role of neutral endopeptidase EC 24.11 is to reduce the activity of secreted GRP-like peptides (Shipp et al, 1991). Decreased EC 24.11 expression in prostatic cancer cell lines has been correlated with increased growth response to bombesin, as well as progression to an androgen-independent phenotype (Papandreou et al, 1998).

Given the reports that GRP is degraded locally in both normal and neoplastic tissues and the mounting evidence that GRP-like peptides may act as growth factors in human breast cancer, we

*Received 2 March 1998*
*Revised 16 June 1998*
*Accepted 17 June 1998*

*Correspondence to:* D Burns, School of Biomedical Sciences, University of Ulster at Jordanstown, Shore Rd, Newtownabbey, Co. Antrim BT37 0QB, Northern Ireland have investigated the possibility that these cells also possess neutral endopeptidase on their cell surface. Here, we report the presence of an EC 3.4.24.11-like enzyme on the surface of breast cancer cells. The growth of these cells and their response to bombesin was also greatly enhanced in the presence of the EC 3.4.24.11 selective inhibitor phosphoramidon.

### MATERIALS AND METHODS

### Materials

Dulbecco's modified Eagle medium (DMEM), RPMI-1640 and L-15 media were obtained from Flow Laboratories, Irvin, UK; fetal calf serum (FCS) and trypsin were purchased from Gibco, Paisley, UK. Phosphoramidon, anti-rabbit immunoprecipitation reagent, 3-(4,5-dimethylthiazole-2-yl)-2,5-diphenyl tetrazolium bromide (MTT), leucine aminopeptidase and the enzyme inhibitors isopropyl difluorophosphate, iodoacetamide and pepstatin were obtained from Sigma Chemical Co, Poole, Dorset, UK. The fluorogenic substrate *N*-succinyl-Ala-Ala-Phe-AMC (AAP-AMC) and bombesin were obtained from Novabiochem, Nottingham, UK. The specific elastase inhibitor was synthesized according to the method previously published (Oleksyszyn and Powers, 1991).

### Cell culture

The T47D (Keydar et al, 1979), ZR-75-1 and MDA-MB-436 breast cancer cell lines were obtained from the European Collection of Animal Cell Cultures, Porton Down, UK. The SCLC H345 cell line was obtained from Imperial Cancer Research Fund Laboratories, London, UK. The T47D and H345 cell lines were maintained in DMEM supplemented with 10% FCS and 10% heat-inactivated FCS respectively. The ZR-75-1 cells were maintained in RPMI-1640 medium supplemented with 5% FCS, and the MDA-MB-436 cells were maintained in L-15 medium supplemented with 10% FCS. Cells were routinely passaged when subconfluent using 2 ml of 2.5 mg $ml^{-1}$ trypsin in calcium-free saline (0.14 M sodium chloride, 5 mM potassium chloride, 4 mM sodium hydrogen carbonate, 5 mM glucose).

### Growth studies

After trypsinization, cells were inoculated into 96-well plates at $1 \times 10^4$ cells per well in medium used for routine passaging.

After 24 h, the medium was removed and the treatments added in medium containing heat and dextran-coated charcoal-treated FCS (HT and DCC FCS) prepared as detailed previously (Nelson et al, 1987, 1991). Treatments were either control (medium only), 0.001–100 nM bombesin or 0.001–100 nM bombesin in the presence of phosphoramidon (0.1–100 ng $ml^{-1}$). For cell enumeration, a modification of the MTT assay (Twentyman and Luscombe, 1987) was used as follows; 10 μl of 3-(4,5-dimethylthiazole-2-yl)-2,5-diphenyl tetrazolium bromide (MTT 20 mg $ml^{-1}$) was added to each well and incubated for 2 h at 37°C, the medium was removed and the formazan crystals were solubilized in 0.2 ml of DMSO. The colour intensity was then measured at 510 nm using a Titertek Multiscan plate reader.

### Preparation of cells and conditioned media for radioimmunoassay (RIA)

Preconfluent flasks (90%) were incubated in serum-free media for 24 h, the conditioned medium was removed and centrifuged at 2500 ***g*** for 10 min and stored at –20°C before Sep-Pak extraction. Cells were removed from monolayer by scraping and washed in serum-free medium and frozen at –20°C. The extraction procedure used for the cell pellets is a modification of that described previously by Gaudino et al (1986). Cells were washed in ice-cold extraction buffer (50 mM Hepes, 5 mM magnesium chloride, 1 mg $ml^{-1}$ bacitracin and 10 μg $ml^{-1}$ phosphoramidon) and boiled for 10 min in 10% formic acid. The extracts were neutralized and clarified by centrifugation at 3800 ***g*** for 15 min. The supernatants and conditioned media were desalted and concentrated using Sep-Pak C18 elution as follows; cartridges were pre-wet with 10 ml each of water, ethanol and 0.05% trifluoroacetic acid (TFA), the sample was passed through the cartridge three times. After washing with 0.05% TFA, the peptides were eluted with 80% acetonitrile containing 0.05% TFA. Positive controls in which [$^{125}$I]GRP was added to cell pellets showed a 79% recovery of radioligand from the cartridge. The eluent was lyophilized and frozen. Immediately before measurement, the residue was dissolved in RIA assay buffer.

### HPLC analysis of cell extracts

Reverse phase high-performance liquid chromatography (HPLC) analysis was carried out using Delta-Prep System (Waters Associates) fitted with a μBondpak $C_{18}$ analytical column. Buffer A was deionized water containing 0.05% trifluoroacetic acid, initially at 98% decreased to 0% after 25 min. Buffer B comprised acetonitrile containing 0.05% trifluoroacetic acid, started at 2% and increased to 65% after 25 min. The solvent flow rate was 1 ml $min^{-1}$ and samples were collected over a period of 1 min. Three runs were performed and eluents for corresponding intervals were pooled and lyophilized and stored at –20°C before radioimmunoassay.

### Radioimmunoassay

This assay utilized a rabbit $GRP_{18–27}$ antiserum raised by this group and described previously (Walker et al, 1995). Five thousand c.p.m. of [$^{125}$I]GRP at a 1:1000 dilution of the antisera and standards or unknown samples were added in duplicate to siliconized Eppendorf tubes in a final volume of 0.2 ml of radio-immunoassay buffer (50 mM sodium phosphate, 10 mM EDTA and 0.3% RIA-grade bovine serum albumin, pH 7.4). A blank (no antiserum) and a standard curve with 0–1000 fmol per tube $GRP_{18–27}$ was set up for each test. After 24 h incubation at 4°C, bound radioligand was immunoprecipitated by anti-rabbit immunoprecipation reagent for 2 h at room temperature and centrifuged at 7500 ***g*** for 15 min. The supernatant was removed and bound radioligand was counted for 1 min in a LBK gamma-counter.

### Fluorogenic enzyme assay

EC 24.11 activity was measured in whole-cell suspensions in a coupled assay using *N*-succinyl-Ala-Ala-Phe 7-amido-4-methyl-coumarin (AAP-AMC) as substrate. Cells were removed from flasks by scraping, washed in phosphate-buffered saline (PBS) and suspended in fluorogenic assay buffer (0.1 M sodium phosphate buffer, pH 7.5, with 0.05% Brijj) at $1 \times 10^{-4}$ cells $ml^{-1}$ before experiment. Thirty-six units (140 U $mg^{-1}$ protein) of leucine aminopeptidase (EC 3.4.11.1) from porcine kidney and *N*-succinyl-Ala-Ala-Phe-AMC (AAP-AMC) were added to the

© Cancer Research Campaign 1999

cuvette to give a final concentration of 10 μM in a final volume of 1.0 ml. The reaction was initiated by the addition of an appropriate number of whole cells in fluorogenic assay buffer. Samples were excited at 383 nm and emission was measured at 455 nm. Production of fluorogenic product at room temperature was measured for 10 min using a Perkin-Elmer fluorimeter model LS50B. Specificity of the assay was tested by performing the following negative controls before each experiment: AAP-AMC alone; AAP-AMC and leucine aminopeptidase; and AAP-AMC with leucine aminopeptidase and cells suspension. Inhibitors were incubated in the presence of cells for 30 min before addition of AAP-AMC and leucine aminopeptidase and the assay.

### Statistical analysis

Control and treatment data from the growth studies were compared using an unpaired Student's *t*-test.

### RESULTS

### Measurement of GRP-LI in breast cancer cell lines

RIA was performed on the breast cancer cell lines T47D, ZR-75-1 and MDA-MB-436 cell pellets and their conditioned medium. The T47D cells were found to contain 1.09 pmol GRP-LI per $10^7$ cells and dilutions paralleled the standard curve (Figure 1). Conditioned medium from the T47D cells, however, did not contain any GRP-LI. Immunoreactivity was not detected in ZR-75-1, MDA-MB-436, Cos-7 or A431 cell lines or their conditioned medium.

The immunoreactivity detected in the T47D cells was further investigated using reversed-phase HPLC. A minor peak eluted after 6 min, however the vast majority eluted in a sharp peak after 20 min. This corresponds very closely with the elution time of 19.5 min for $GRP_{18-27}$ (Figure 2).

### Effect of phosphoramidon on BN-induced cell growth

MTT assays were set up to examine the effect of 0.1–100 ng $ml^{-1}$ phosphoramidon alone and in combination with 0.1 nM BN on the T47D cells in HT and DCC FCS (Figure 3). After 3 days, 1–100 ng $ml^{-1}$ of the inhibitor gave a marked inhibition (19–35%) of cell growth alone. This dose range of phosphoramidon also dramatically potentiated the response of these cells to BN. Whereas BN alone showed a small increase in cell number after 3 days, in this instance it was not statistically significant. However, in combination with 0.1–100 ng $ml^{-1}$ phosphoramidon the cell growth was markedly increased. Phosphoramidon (0.1 ng $ml^{-1}$ and 1 ng $ml^{-1}$) gave a 38% and 30% increase in BN-induced growth respectively. Phosphoramidon (0.1 ng $ml^{-1}$) appeared to be a threshold dose above which higher concentrations of inhibitor produced no increased effect. When compared with cell numbers in the presence of 1 ng $ml^{-1}$ phosphoramidon alone, 0.1 nM BN in combination with phosphoramidon produced an increased growth of 132% ($P < 0.01$).

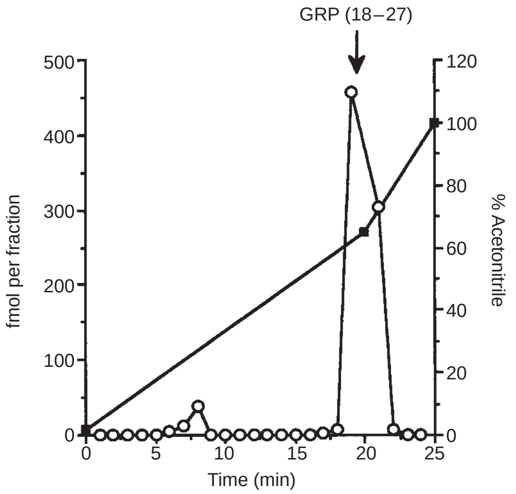

**Figure 2** Reverse-phase HPLC separation of T47D cell extract followed by GRP radioimmunoassay of fractions. Elution buffer was acetonitrile containing 0.05% trifluoroacetic acid starting at 2% and increasing to 65% after 25 min. Eluents from corresponding fractions were pooled from three runs and lyophilized before radioimmunoassay. Elution time of GRP $_{18-27}$ is indicated by an arrow

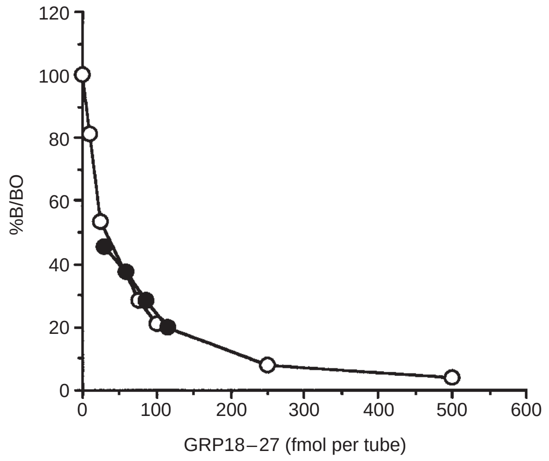

**Figure 1** Radioimmunoassay standard curve for GRP $_{18-27}$ –○– and T47D cell extract –●–. [125I]GRP (5000 c.p.m.) and antiserum at final dilution of 1:1000 per tube were used and incubations were performed at 4°C for 24 h. Data points represent the means of duplicate tubes

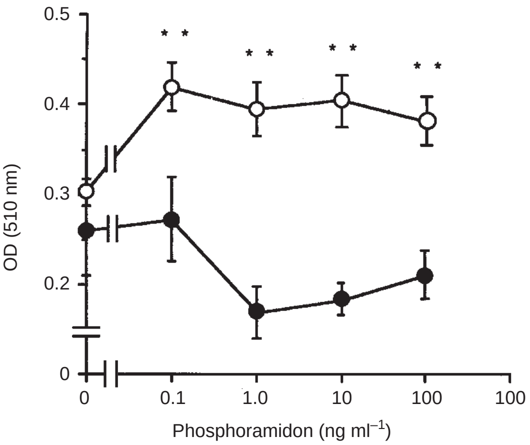

**Figure 3** The effect of phosphoramidon –●–, and phosphoramidon in the presence of bombesin (0.1 nM) –○– on the growth of T47D cells after 3 days in DMEM +10% HT and DCC-treated FCS. Results are the mean optical density + s.d. for eight wells. $^{**}P < 0.01$ vs phosphoramidon only in Student's *t*-test

 *© Cancer Research Campaign 1999*

### Fluorogenic measurement of EC 3.4.24.11

For all experiments, the substrate alone produced baseline fluorescence indicating that there was no autohydrolysis. Similarly, the substrate in the presence of leucine aminopeptidase (LAP) produced baseline fluorescence eliminating the possibility of enkephalinase contamination of the LAP. The SCLC cell line H345 was found to produce a turnover of substrate of 303.6 pmol per $10^6$ cells $h^{-1}$. This activity was reduced by 76.6% in the presence of phosphoramidon. The T47D cells exhibited a much lower turnover of substrate with 4.6 pmol per $10^6$ cells $h^{-1}$ being recorded alone with 56% of this activity being phosphoramidon sensitive. The inhibitors DFP (0.5 mM), iodoacetamide (10 μM) and pepstatin (10 μM) did not inhibit substrate turnover. In the presence of the elastase inhibitor Z-Val-diphenyl phosphonate (43 μM), a small inhibition was recorded (data not shown).

### DISCUSSION

It is widely accepted that SCLC tumours arise from endocrine cells because they display characteristics such as amine precursor uptake and decarboxylation (APUD) staining and contain several neuropeptides (Baylin et al, 1980; Gazdar et al, 1980; Marangos et al, 1982). Some of these neuropeptides, including bombesin, have been shown to act as autocrine growth factors in numerous SCLC cell lines (Cuttitta et al, 1985; Bepler et al, 1988; Sethi and Rozengurt, 1991). Unlike SCLC, the vast majority of breast cancers are epithelium-derived tumours, with only 5% being estimated to be carcinoids with a neuroendocrine origin (Papotti et al, 1989). These tumours are reported to be strongly immunoreactive for GRP-LI (Nesland et al, 1985; Pagini et al, 1991). The presence of neuropeptides in a large proportion of breast tumours would, therefore, be unexpected, however, in at least one study mRNA for GRP was detected in 25% of unselected breast tumours and it had poor correlation with neuroendocrine differentiation (Pagini et al, 1991). Interestingly, GRP-LI has also been reported in breast milk in a number of studies (Ekman et al, 1985; Westrom et al, 1987). Thus, the presence of GRP in breast cancer cells may instead be due to lactational differentiation, a phenomenon which is known to occur in breast carcinomas (Clayton et al, 1982; Lee et al, 1984; Baildam et al, 1988).

The occurrence of GRP-LI in breast cancer cell lines is not without controversy. Although it has been detected in T47D cells in this study and by Vangsted et al (1991), Northern blot analysis for prepro-GRP mRNA has been negative (Giachetti et al, 1990; Pagini et al, 1991). This lack of correlation between GRP immunoreactivity and mRNA detection is observed in other tumour types (Hamid et al, 1987, 1989), and has been attributed to antisera cross-reactivity with related neuropeptides (Pagini et al, 1991). Alternatively, detection of a low-abundance mRNA for this peptide may require reverse transcription polymerase chain reaction (RT-PCR) and this was not carried out in the studies on breast cancer cell lines (Giachetti et al, 1990; Pagini et al, 1991). Hence, it is important to further characterize this immunoreactivity. In this study, HPLC analysis strongly suggests the major peak is $GRP_{18-27}$ because it co-eluted with this standard. This is in agreement with similar chromatographic analysis performed by Vangsted et al (1991). The minor, more hydrophilic peak may represent a larger peptide, perhaps GRP. Failure to detect GRP-LI in conditioned medium from T47D cell-conditioned medium here and by Vangsted et al (1991) suggests that it is rapidly cleared from the extracellular surface. Of importance is the fact that the C-terminal extension peptide (CTEP), which is cleaved from prepro-GRP during processing, has been found in the T47D-conditioned medium in the absence of the mature peptide (Vangsted et al, 1991). Accumulation of a similar stable CTEP has also been described in SCLC cell lines (Vangsted and Schwartz, 1990). The processing and subsequent degradation of the prepro-GRP moiety clearly happens extracellularly in both cancer cell types.

Inhibition of fluorogenic substrate hydrolysis by phosphoramidon is a well-documented specific test for EC 3.4.24.11 activity (Hudgin et al, 1981; Shipp et al, 1991; Frame et al, 1996). The demonstration that T47D whole cell-mediated turnover of AAP-AMC is sensitive to phosphoramidon strongly suggests the presence of the membrane-bound endopeptidase EC 3.4.24.11 on these cells. In the growth studies, after 3 days all concentrations of phosphoramidon tested (0.1–100 ng $ml^{-1}$) enhanced BN-induced growth. At this time, 1–100 ng $ml^{-1}$ phosphoramidon alone inhibited growth, however the reason for this is unclear at present. In other tissues, EC 3.4.24.11 has been shown to degrade a range of peptides including enkephalins, substance P, bradykinin, endothelin and angiotensin I and II (Matsas et al, 1984; Vijayaraghavan et al, 1990). Although only endothelin (which is not mitogenic) has been shown to be produced by the T47D cells (Schrey et al, 1992), the possibility of other as yet unidentified EC 3.4.24.11-labile growth factors being expressed by the T47D cells cannot be ruled out. Previous work in this laboratory found BN to be mitogenic for breast cancer cell lines in vitro, with 0.001–0.1 nM giving significant growth stimulation in the T47D cells (Nelson et al, 1991). The potentiation of BN-induced growth by phosphoramidon after 3 days is likely to be due to a prolonged half-life of the peptide in the medium.

The fluorogenic assay found that there was approximately 100-fold more EC 3.4.24.11 activity on the H345 SCLC cell line compared with the T47D cells. This may be an explanation for the difference in BN dose required for mitogenesis in cell lines from these two cancer types. GRP/BN-stimulated colony formation is maximal at 50 nM in SCLC cells, but in the breast cancer cells significant stimulation is observed in the picomolar range in this laboratory (Carney et al, 1981; Nelson et al, 1991). However, this variation in mitogenicity may also be due to differing numbers of BN/GRP receptors on these cell lines. Variations in the mitogenic response to BN/GRP have been also observed between the classical and variant SCLC cell lines, this may be due to differing numbers of GRP receptors reported on these cell lines (Kado-Fong and Malfoy, 1989; Cardona et al, 1991). Stimulation of growth of breast cancer cells by BN has also been confirmed by other workers under serum-free or HT and DCC FCS conditions. In all these studies, no effect was found in medium supplemented with untreated FCS. A soluble form of EC 3.4.24.11 has been described in urine, plasma and breast cyst fluid, but routine culture FCS to date has not been investigated. Although the lack of response to BN in untreated FCS has been attributed to the presence of endogenous GRP-LI which is removed by HT and DCC treatment, the role of a soluble EC 3.4.24.11 in FCS is yet to be studied.

These findings have practical implications for experimental research in bombesin-like peptides and breast cancer. Firstly, failure to inhibit EC 24.11-like activity in the past may have compromised RIA or immunocytochemistry sensitivity, however all the buffers used for cell homogenization for RIA and HPLC in this study contained phosphoramidon. Secondly, binding of ligands in radioreceptor studies on breast cancer cell lines could be signifi-

© Cancer Research Campaign 1999

cantly reduced because phosphoramidon was found to greatly increase binding of [$^{125}$I]GRP to SCLC cells (Cardona et al, 1992).

Extensive research efforts have been employed in the development and evaluation of GRP receptor antagonists as potential treatments for SCLC and, more recently, pancreatic, prostatic, gastric, colorectal and breast cancer (Jungwirth et al, 1997; Nagy et al, 1997). The presence of EC 24.11-like activity on breast cancer cells is a crucial consideration in the design of GRP receptor antagonists. Whereas the sissile bonds and the relative rates of hydrolysis have been identified for GRP and its homologues, to date none of these antagonists have been evaluated for resistance to cleavage by the enzyme.

EC 24.11 has been described on human endometrium, where levels vary through the menstrual cycle but activity correlates with plasma progesterone levels (Casey et al, 1991; Head et al, 1993). More recently, reduced EC.24.11 mRNA expression has been demonstrated on endometrial cancer cells compared with normal endometrial tissue (Pekonen et al, 1995). Levels of this enzyme are also reported to be 70–90% reduced on SCLC cell lines (Shipp et al, 1991). Additionally, EC 24.11 loss appears to play a role in the development of androgen-independent cells by allowing neuropeptides such as GRP to enhance growth (Papandreou et al, 1998). Thus, reduced clearance of peptides may be the aberrant factor which results in increased growth response of cancer cells. Therefore, the concept of measuring peptidases in tumours may be complementary to assessing growth factor levels as indicators of tumour growth or progression.

This is the first indication of the presence of such an enzyme on human breast cancer cells and supports the finding that GRP is an autocrine growth factor in the T47D cells. It is possible that reduced activity of this enzyme accompanies breast tumorigenesis because levels in breast cysts decreased in those women who carry a higher risk of future breast cancer when compared with those who are not associated with any subsequent increase in incidence (Frame et al, 1996).

In normal tissues, EC 3.4.24.11 has a diverse distribution, but is usually found on cells bearing specific receptors for the multiple possible target peptides. In the brain, it is responsible for the inactivation of the enkephalins (Malfroy et al, 1978), whereas in lung it down-regulates neurogenic inflammation by reducing local levels of tachykinins (Martins et al, 1990; Nadel, 1990; Nadel and Borson, 1991). It is also responsible for control of GRP-induced growth in fetal lung, where alterations in levels of GRP throughout maturation are accompanied by corresponding changes in EC 3.4.24.11 levels (King et al, 1993).

Given that GRP-like peptides are present in high amounts in breast milk, it is probable that an enzyme would also control these levels locally. EC 3.4.24.11 may also be present on normal breast cells in a similar manner to normal lung tissue as Gusterson et al (1985) reported specific staining for the CALLA antigen on normal human breast sections. This antigen has subsequently been shown to be identical to EC 3.4.24.11 (Ship et al, 1989). It, therefore, would be of interest to determine whether EC 3.4.24.11 or GRP-like peptides played a modulatory role in normal breast development.

In conclusion, this study demonstrates the production of a $GRP_{18–27}$ peptide by T47D breast cancer cells, but not by ZR-75-1 or MDA-MB-436. This peptide is rapidly processed, degraded and inactivated in the extracellular medium. A previously unreported enzyme with an EC 3.4.24.11 profile on the human breast cancer cell line T47D is also detected. Although this enzyme is present at low levels, it significantly modulates the growth of the T47D cells both alone and in response to BN in vitro.

### ACKNOWLEDGEMENTS

The authors gratefully acknowledge the financial assistance of the Association for International Cancer Research, Action Cancer Northern Ireland and the Department of Education for Northern Ireland.

### REFERENCES

Baildam AD, Howell A, Barnes DM, Redford J, Healey K, Swindell R and Sellwood RA (1988) Expression of differentiation antigens within human mammary tumours is related to response to endocrine therapy and survival. *Int J Cancer* **42**: 154–158

Baylin SB, Abeloff MD, Goodwin G, Carney DN and Gazdar AF (1980) Activities of L-dopa decarboxylase and diamine oxidase (histaminase) in human lung cancers and decarboxylase as a marker for small (oat) cell cancer in cell culture. *Cancer Res* **40**: 1990–1994

Bepler G, Rotsch M, Jacques G, Haeder M, Heymanns J, Hartogh G, Kiefer P and Havemann K (1988) Peptides and growth factors in small cell lung cancer – production, binding sites and growth effects. *J Cancer Res Clin Oncol* **114**: 235–244

Bostwick DG and Bensch KG (1985) Gastrin-releasing peptide in human neuroendocrine tumours. *J Pathol* **147**: 237–244

Bostwick DG, Roth KA, Evans CJ, Barchas JD and Bensch KG (1984) Gastrin-releasing peptide, a mammalian analog of bombesin, is present in human neuro-endocrine lung-tumours. *Am J Pathol* **117**: (2) 195–200

Bunnett NW, Turner AJ, Hryszko J, Kobashi R and Walsh AJ (1988) Isolation of endopeptidase 24.11 (EC 3.4.24.11, enkephalinase) from the pig stomach – hydrolysis of substance P, gastrin-releasing peptide 10. *Gastroenterology* **95**: 952–557

Cardona C, Rabbitts PH, Spindel ER, Ghatei MA, Bleehen NM, Bloom SR and Reeve JG (1991) Production of neuromedin B and neuromedin B gene expression in human lung tumour cell lines. *Cancer Res* **51**: 5205–5211

Cardona C, Bleehen NM and Reeve JG (1992) Characterisation of ligand-binding and processing by gastrin-releasing peptide receptors in a small-cell lung cancer cell line. *Biochem J* **281**: 115–120

Carney DN, Bunn Jr PA, Gazdar AF, Pagan JA and Minna JD (1981) Selective growth in serum-free hormone-supplemented medium of tumour cells obtained by biopsy from patients with small cell carcinoma of the lung. *Proc Natl Acad Sci USA* **78**: 3185–3189

Casey ML, Smith JW, Nagai K, Herch LB & MacDonald PC (1991) Progesterone-regulated cyclic modulation of membrane metalloendopeptidase (enkephalinase) in human endometrium. *J Biol Chem* **266**: 23041–23047

Clayton F, Ordonez NG, Sibley RK and Hanssen G (1982) Argyrophilic breast carcinomas – evidence of lactational differentiation. *Am J Surg Pathol* **6**: 323–333

Cuttitta F, Carney DN, Mulshine J, Moody TW, Fedorko J, Fischler A and Minna JD (1985) Bombesin-like peptides can function as autocrine growth-factors in human small-cell lung cancer. *Nature* **316**: 823–826

Ekman R, Ivarsson S and Jansson L (1985) Bombesin, neurotensin and pro-γ melanotropin immunoreactants in human milk. *Regul Peptides* **10**: 99–105

Erspamer V, Falconierri-Erspamer G and Inselvini M (1970) Some pharmacological actions of alytesin and bombesin. *J Pharm Pharmacol* **22**: 875–876

Erspamer V, Falconierri-Erspamer G, Inselvini M and Negri L (1972*a*) Occurrence of bombesin and alytesin in extracts of the skin of three European discoglossid frogs and pharmacological actions of bombesin on extravascular smooth muscle. *Br J Pharmacol* **45**: 333–348

Erspamer V, Mechiorri P and Soprazi N (1972*b*) Action of bombesin on the systemic arterial blood pressure of some experimental animals. *Br J Pharmacol* **45**: 442–450

Foster CS and Tan KS (1984) Paracrine differentiation in human primary breast carcinomas (abstract). *Regul Peptides* **9**: 329

Frame KL, Patton K, Reed MJ, Ghilchik MW and Parish DC (1996) Angiotensin-converting enzyme and enkephalinase in human breast cyst fluid. *Br J Cancer* **74**: 807–813

Gaudino G, Debortoli M and Lazarus LH (1986) A bombesin-related peptide in experimental mammary tumours in rats. *Ann NY Acad Sci* **464**: 450–453

© Cancer Research Campaign 1999

Gazdar AF, Carney DN, Russel EK, Sims HL, Baylin SB, Bunn PA Jr, Guccion JG and Minna JD (1980) Establishment of continuous clonal cultures of small-cell carcinoma of the lung which have amine precursor uptake and decarboxylation cell properties. *Cancer Res* **40**: 3502–3507

Giacchetti S, Gauville C, Cremoux De P, Bertin L, Berthon P, A bita JP, Cuttitta F and Calvo F (1990) Characterisation, in some human breast cell lines, of gastrin-releasing peptide-like receptors which are absent in normal breast epithelial cells. *Int Cancer J* **46**: 293–298

Gusterson BA, Monaghan P, Mahendran R, Ellis J and O'Hare MJ (1985) Identification of myoepithelial cells in human and rat breasts by anti-common acute lymphoblastic leukemia antigen antibody A12. *J Natl Cancer Inst* **77**: 343–349

Hamid QA, Addis BJ, Springall DR, Ibrahim NBN, Ghatei MA, Bloom SR and Polak JM (1987) Expression of the C-terminal peptide of human pro-bombesin in 361 lung endocrine tumours, a reliable marker and possible prognostic indicator for small cell carcinoma. *Virchows Arch A* **411**: 185–192

Hamid QA, Bishop AE, Springall DR, Adams C, Giaid DX, Ghatei MA, Legon S, Cuttitta F, Rode J, Spindel ER and Bloom SR (1989) Detection of human pro-bombesin mRNA in neuroendocrine (small cell) carcinoma of the lung. *Cancer* **63**: 266–271

Head JR, MacDonald PC and Casey ML (1993) Cellular localisation of membrane metalloendopeptidase (enkephalinase) in human endometrium during the ovarian cycle. *J Clin Endocrinol Metab* **76**: 769–776

Hudgin RL, Charleson SE, Zimmerman M, Mumford R and Wood PL (1981) Enkephalinase – selective peptide inhibitors. *Life Sci* **29**: 2593–2601

Jungwirth A, Pinski J, Galvin J, Halmos G, Szepeshazi K, Cai RZ, Groot K, Vadillo-Buenfil M and Schally AV (1997) Inhibition of growth of androgen-independent DU-145 prostate cancer *in vivo* by luteinising hormone-releasing hormone antagonist cetrorelix and bombesin antagonists RC-3940-II and RC-3950-II. *Eur J Cancer* **33**: 1141–1148

Kado-Fong H and Malfroy B (1989) Effects of bombesin on human small cell lung cancer cells – evidence for a subset of bombesin non responsive cell lines. *J Cell Biochem* **40**: 431–437

Keydar I, Chen L, Karby S, Weiss FR, Delarea J, Radu M, Chaicik S and Brenner HJ (1979) Establishment and characterisation of a cell line of human breast carcinoma origin. *Eur J Cancer* **15**: 659–670

King KA, Hua J, Torday JS, Drazen JM, Graham SA, Shipp MA and Sunday ME (1993) CD10/neutral endopeptidase 24.11 regulates foetal lung growth and maturation *in utero* by potentiating endogenous bombesin-like peptides *in utero*. *J Clin Invest* **91**: 1969–1973

Lee AK, Delellis RA, Rosen PP, Herbert-Stanton T, Tallberg K, Garcia C and Wolfe HJ (1984) Alpha-lactalbumin as an immunohistochemical marker for metastatic breast carcinomas. *Am J Surg Pathol* **8**: 93–100

Malfroy B, Swerta JB, Guyon A, Roques BP and Swartz JC (1978) High-affinity enkephalin-degrading peptidase in brain is increased after morphine. *Nature (London)* **276**: 523–526

Marangos PJ, Gazdar AF and Carney DN (1982) Neuron specific enolase in human small cell-carcinoma cultures. *Cancer Lett* **15**: 67–71

Martins MA, Shore SA, Gerard NP, Gerard C and Drazen JM (1990) Peptidase modulation of the pulmonary effects of tachykinins in tracheal superperfused guinea-pig lungs. *J Clin Invest* **85**: 170–176

Matsas R, Kenny AJ and Turner AJ (1984) The metabolism of neuropeptides – the hydrolysis of peptides, including enkephalins, tachykinins and their analogs, by endopeptidase 24.11. *Biochem J* **223**: 433–440

McDonald TJ, Jornvall H, Nilsson G, Vagne M, Ghatei M, Bloom SR and Mutt V (1979) Characterisation of a gastrin releasing peptide from porcine non-antral gastric tissue. *Biochem Biophys Res Commun* **90**: 227–233

McDonald TJ, Jornvall H, Ghatei M, Bloom SR and Mutt V (1980) Characterisation of an avian gastric (proventricular) peptide having sequence homology with the porcine gastrin-releasing peptide and the amphibian peptides bombesin and alytensin. *FEBS Lett* **122**: 45–48

McKillop JM, Carragher A, Johnston CF, Murphy RF and Buchanan KD (1988) Identification of GRP in a small population of human breast carcinomas. *Regul Peptides* **22**: 420

Minamino N, Kangawa K and Matsuo H (1984) Neuromedin-B is a major bombesin-like peptide in rat-brain – regional distribution of neuromedin-B and neuromedin-C in rat-brain, pituitary and spinal cord. *Biochem Biophys Res Commun* **124**: 925–932

Moody TW, Thoa NB, O'Donohue T and Pert C (1980) Bombesin-like peptides in rat brain: localisation in synaptosomes and release from hypothalmic slices. *Life Sci* **26**: 1707–1712

Moody TW, Bertness V and Carney DN (1983) Bombesin-like peptides and receptors in human tumour cell lines. *Peptides* **4**: 683–686

Nadel JA (1990) Decreased neutral endopeptidases: possible role in inflammatory diseases of airways. *Lung* (**suppl.**): 123–127

Nadel AJ and Borson DB (1991) Modulation of neurogenic inflammation by neutral endopeptidase. *Am Rev Respir Dis* **143** (suppl.): S33–S36

Nagalla SR, Gibson BW, Tang DZ, Reeve JR and Spindel ER (1992) Gastrin-releasing peptide (GRP) is not mammalian bombesin – identification and molecular cloning of a true amphibian GRP distinct from mammalian bombesin in *Bombina orientalis*. *J Biol Chem* **267**: 6916–6922

Nagy A, Armatis P, Cai RZ, Szepeshazi K, Halmos G and Schally AV (1997) Design, synthesis and in vitro evaluation of cytotoxic analogs of bombesin-like peptides containing doxorubicidin or its intensely potent derivative, 2-pyrrolinodoxorubicidin. *Proc Natl Acad Sci USA* **94**: 652–656

Nelson J, Clarke R, McFerran NV and Murphy RF (1987) Morphofunctional effects of phenol red on oestrogen-sensitive breast cancer cell lines. *Biochem Soc Trans* **15**: 244

Nelson J, Donnelly M, Walker B, Gray J, Shaw C and Murphy RF (1991) Bombesin stimulates proliferation of human breast cancer cells in culture. *Br J Cancer* **63**: 933–936

Nesland JM, Memoli VA, Holm R, Gould VE and Johannessen JV (1985) Breast carcinomas with neuroendocrine differentiation. *Ultrastruct Pathol* **8**: 225–240

Oleksyszyn J and Powers JC (1991) Irreversible inhibition of serine proteases by peptide derivatives of (alpha-aminoalkyl) phosphonate diphenyl esters. *Biochemistry* **30**: 485–493

Orloff MS, Reeve Jr JR, Ben-Avram CM, Shively JE and Walsh JH (1984) Isolation and sequence-analysis of human bombesin-like peptides. *Peptides* **5**: 865–870

Pagani A, Papotti M, Sanfilippo B and Bussolati G (1991) Expression of the gastrin-releasing peptide gene in carcinomas of the breast. *Int J Cancer* **47**: 371–375

Papandreou CN, Usami B, Geng Y, Bogenrieder T, Freeman R, Wilk S, Finstad CL, Reuter VE, Powell CT, Scheinberg D, Magill C, Scher HI, Albino AP and Nanus DM (1998) Neutral endopeptidase 24.11 loss in metastatic human prostate cancer contributes to androgen-independent progression. *Nature Med* **4**: 50–57

Papotti M, Macri L, Bussoliti G and Reubi JC (1989) Correlative study on neuroendocrine differentiation and presence of receptors in breast carcinomas. *Int J Cancer* **43**: 365–369

Pekonen P, Nyman T, Ammala M and Rutmanen EM (1995) Decreased expression of messenger RNAs encoding endothelin receptors and neutral endopeptidase 24.11 in endometrial cancer. *Br J Cancer* **71**: 59–63

Reeve JR, Walsh JH, Chew P, Clark B, Hawke D and Shively JE (1983) Amino acid sequences of 3 bombesin-like peptides from canine intestine extracts. *J Biol Chem* **258**: 5582–5588

Relton JM, Gee NS, Matsas R, Turner AJ and Kenny AJ (1983) Purification of endopeptidase-24.11 (enkephalinase) from pig brain by immunosorbent chromatography. *Biochem J* **215**: 519–523

Schrey MP, Patel KV and Tezapsidis N (1992) Bombesin and glucocorticoids stimulate human breast cancer cells to produce endothelin, a paracrine mitogen for breast stromal cells. *Cancer Res* **52**: 1786–1790

Sethi T and Rozengurt E (1991) Multiple neuropeptides stimulate clonal growth of small-cell lung cancer – effects of bradykinin, vasopressin, cholecystokinin, galinin and neurotensin. *Cancer Res* **51**: 3621–3623

Shipp MA, Vijayaraghavan J, Schmidt EV, Masteller EL, D'Adamido L, Hersh LB and Reinherz EL (1989) Common acute lymphoblastic leukemia antigen (CALLA) is active neutral endopeptidase 24.11 ('enkephalinase'): direct evidence by cDNA transfection analysis. *Proc Natl Acad Sci USA* **86**: 297–301

Shipp MA, Tarr GE, Chen CY, Switzer SN, Hersh LB, Stein H, Sunday ME and Reinherz EL (1991) CD10/neutral endopeptidase 24.11 hydrolyses bombesin-like peptides and regulates the growth of small cell carcinomas of the lung. *Proc Natl Acad Sci USA* **88**: 10662–10666

Sunday ME, Kaplan LM, Mototoma E, Chin WW and Spindel E (1988) Gastrin-releasing peptide (mammalian bombesin) gene expression in health and disease. *Lab Invest* **59**: 5–24

Twentyman PR and Luscombe M (1987) A study of some variables in a tetrazolium dye (MTT) based assay for cell growth and chemosensitivity. *Br J Cancer* **56**: 279–285

Vangsted A and Schwartz TW (1990) Production of gastrin-releasing peptide ($_{18–27}$) and a stable fragment of its precursor in small cell lung carcinoma cells. *J Clin Endocrinol Metab* **70**: 1586–1593

Vangsted AJ, Andersen EV, Nedergaard L and Zeuthen J (1991) Gastrin-releasing peptide GRP (14–27) in human breast cancer cells. *Breast Cancer Res Treatment* **19**: 119–128

Vijayaraghavan J, Scicili AG, Carretero OA, Slaughter C, Moonaw C and Hersh LB (1990) The hydrolysis of endothelins by neutral endopeptidase 24.11 (encephalinase). *J Biol Chem* **265**: 14150–14155

Walker B, Gray J, Burns DM, Wang W, Adrain T, Nichols H, Murphy RF and Nelson J (1995) Carboxyfluorescein and biotin neuromedin C analogues: synthesis and applications. *Peptides* **16**: 255–261

© Cancer Research Campaign 1999

Weber CJ, O'Dorisio TM, McDonald TJ, Howe B, Koschitzky T and Merriam L (1989) Gastrin-releasing peptide-like, calcitonin gene-related peptide-like and calcitonin-like immunoreactivity in human breast cyst fluid and gastrin-releasing peptide-like immunoreactivity in human breast carcinoma cell lines. *Surgery* **106**: 1134–1139

Westrom BR, Ekman R, Svenson L, Svensden J and Karlsson W (1987) Levels of immunoreactive insulin, neurotensin and bombesin in porcine colostrum and milk. *J Paediatr Gastroenterol Nutr* **6**: 460–465

Wood SM, Wood JR, Ghatei MA, Lee YC, O'Shaughnessy D and Bloom SR (1981) Bombesin, somatostatin and neurotensin-like immunoreactivity in bronchial carcinoma. *J Clin Endocrinol Metab* **53**: 1310–1312

Yamaguchi K, Abe K, Kameya T, Adachi I, Taguchi S, Otsubo K and Yanaihara N (1983) Production and molecular-size heterogeneity of immunoreactive gastrin-releasing peptide in foetal and adult lungs and primary lung-tumours. *Cancer Res* **43**: 3932–3939

Yano T, Pinski J, Groot K and Schally AV (1992) Stimulation by bombesin and inhibition by bombesin gastrin-releasing peptide antagonist RC-3095 of growth of human breast cancer cell lines. *Cancer Res* **52**: 4545–4547

Zoeller RT, Lebacq-Verheyden AM and Battey JF (1989) Distribution of 2 distinct messenger ribonucleic acids encoding gastrin releasing peptide. *Peptides* **10**: 415–422

© Cancer Research Campaign 1999
